# Research on the Analysis of Correlation Factors of English Translation Ability Improvement Based on Deep Neural Network

**DOI:** 10.1155/2022/9345354

**Published:** 2022-08-29

**Authors:** Xiaojun Jiang

**Affiliations:** School of Translation Studies, Xi'an Fanyi University, Xi'an, Shaanxi 710105, China

## Abstract

This paper adopts the algorithm of the deep neural network to conduct in-depth research and analysis on the factors associated with the improvement of English translation ability. This study focuses on text complexity, adding discourse complexity features in addition to focusing on lexical and syntactic dimensions, exploring the application of neural network algorithm in the construction of text complexity grading model based on feature optimization, and examining the performance and generalization ability of the model. The rationality of the grading of the material is verified. After determining the model input features and training corpus, different classification algorithms were used to build the models and compare their performance. Meanwhile, compared with the models constructed based on common traditional readability formulas and other single-dimensional features, the models constructed based on the feature set of this study have significant advantages, with 20 to 30 percentage points higher in each performance evaluation index. The pseudo-parallel corpus is constructed, back translation is performed after obtaining the pseudo-parallel corpus, and finally, the data migration effect is measured and recorded on the low-resource Chinese-English parallel corpus and Tibetan-Chinese parallel corpus, and the cycle continues until the model performance is no longer improved. The low-resource neural machine translation model based on model migration learning improved 3.97 and 2.64 BLEU values in the low-resource English translation task, respectively, and reduced the training time; based on this, the data migration learning method further improved 2.26 and 2.52 BLEU values.

## 1. Introduction

Language, as the primary means of human communication, is one of the most important communication tools. With the development of the times and economic globalization, people around the world are communicating and cooperating increasingly frequently, and people in different countries are increasingly closely connected, and language barriers are becoming more serious and obvious, and the need for seamless communication and understanding becomes crucial [1]. Machine translation as an effective means to solve people's cross-linguistic communication barriers has been a hot topic of concern for relevant researchers. With the great progress of deep learning research, Neural Machine Translation (NMT) based on neural networks has gradually emerged. With the rapid development of the Internet, natural language processing has become one of the main development directions of future technology [2]. Machine translation technology, as an important branch of natural language processing, has penetrated our daily life and become the current research hotspot in the field of information processing, the purpose of which is to enable people to reduce the language barriers of communication between different countries and nationalities through machine translation [3]. In this paper, we conduct a series of researches on the existing neural machine translation method under the limited parallel corpus resources. Specifically, firstly, model training is conducted for the Chinese-English parallel corpus with abundant parallel corpus resources, and the model parameters with the best performance of neural machine translation model are studied and trained; then, through the generality of the neural machine translation model, the trained model parameters are transferred to the language pairs with scarce parallel corpus using the idea of transfer learning for model training; finally, how to use the existing language resources to expand the corpus by combining data migration techniques to expand the existing corpus resources and apply them to the monolingual corpus to help the training of neural machine translation models is discussed.

To help readers match the reading text to their language level, researchers have long been trying to build formulas or models that can automatically calculate and estimate the complexity of the text. Automatic text complexity grading is achieved by transforming a target text into a set of complex features and then using these features to compute formulas or train models to automatically predict or grade the complexity of a new text [4]. However, which text features can characterize the text complexity and how these features are associated with the corresponding complexity level have been the questions that researchers have been trying to solve. Currently, most scholars and translation researchers believe that English-Chinese translation competence consists of multiple components, but they have not reached a consensus on the specific components that constitute translation competence [5]. They have proposed many different models of translation competence research and tend to encompass more components into the translation model, which undoubtedly makes translators more confused and unable to identify the core components of translation competence. The goal of translation competence research is to break through this dilemma, clear up the fog for translators, clarify the components of translation competence, better guide translation practice, and promote the improvement of translation quality, which is the same as the research goal of translation writing.

The theory of translation writing takes the practice of English-Chinese translation as the starting point, aiming at the goal of improving translators' translation writing ability by solving the problem of how to translate properly and skillfully. Based on the theoretical achievements of translation writing theory, this paper focuses on the translation process itself and studies the English-Chinese translation ability under the perspective of translation writing theory and firstly concludes that translation ability is translation writing ability; in other words, translation ability consists of various abilities that translators must possess at each stage of the translation writing process [6]. The first two types of reading help translators improve their English language skills and acquire extensive knowledge, which helps translators understand the source text more fully, while the source text reading is fundamental in determining the quality of the translation. To present a high-quality translation, the translator must have the ability to read and understand the source text. In the process of English-Chinese translation and writing, the translator can only acquire translation reading ability and achieve the goal of improving English-Chinese translation ability and presenting the best translation when he or she fully perceives the content, genre, and structure of the source text as well as the style of writing.

## 2. Related Works

Parallel utterances with many domain-specific languages in each domain can be considered high-resource but are poorly resourced in general domains. For example, a common source of parallel statements in resource-poor languages is news, which is translated into hundreds of languages [7]. However, it is domain- and style-specific text, and the translation does not meet people's needs when the system is applied to different domains. In addition to the size of the parallel corpus, in specific language categories, its parallel corpus resources are relatively lacking; in this case, the neural machine translation system for model training and optimization is not effective, and its translation results are difficult to achieve expected results [8]. Hassanpour et al. proposed the so-called attention mechanism. The attention mechanism gives the network the ability to reconsider all input words and use this information when generating new words [9]. The previous architecture, which processed all input words together and therefore made the training and inference process faster, was redesigned with convolutional neural networks. Zhang et al. define the very low-resource scenario by the minimum amount of data needed to obtain a reasonable translation quality by NMT. They found that English ⟶ Romanian translations can be considered for parallel sentences of 13–28k in the case of extremely limited resources. They found that English ⟶ Romanian translation can consider 13–28k parallel sentences with extremely limited resources [10]. Akan et al. proposed a speech band extension method based on Convolutional Neural Network (CNN), which is different from previous speech band extension studies and is the first. The input of the model is the narrowband speech in the time domain using cubic spline interpolation, and the output is the broadband speech reconstructed by the CNN model [11]. In the same year, Google disruptively proposed a neural machine translation model that completely abandoned recurrent neural networks and convolutional neural networks. The model also uses “encoder-decoder” as the framework of the model and employs a multiheaded attention mechanism and feedforward neural networks for the structure of encoder and decoder in the model, and the model has achieved exciting results in machine translation tasks for many language pairs [12].

Speech band extension techniques were first proposed in the 1930s, and the quality of reconstructed wideband speech was not much improved due to the limitations of hardware equipment and signal processing techniques at that time. Until the source-filter model was proposed, the speech band extension process was simplified to excitation signal generation and spectral envelope estimation for describing the vocal tract model according to the speech generation mechanism. Speech band extension techniques can be divided into blind and nonblind [13]. The mainstream solution is blind speech band extension; that is, narrowband speech is processed and restored by the speech band extension algorithm at the receiving end of the communication device. In the training phase, the model input is the log power spectrum of narrowband speech and the output is the high-frequency log spectrum of wideband speech, and the mapping model is trained using the minimum mean square error as the objective function. In the extension phase, the model inputs the log power spectrum of the test narrowband speech and estimates the missing high-frequency phase using the low-frequency phase signal of the test narrowband speech and then performs a discrete Fourier inverse transform to reconstruct the broadband speech signal [14]. This model achieved better results than the Gaussian mixture model in terms of subjective and objective evaluation, but there was a spectral discontinuity problem in the transition segment between the narrowband spectrum and the high-frequency spectrum of the reconstructed wideband speech. Subsequently, Gu and Li solved the spectral discontinuity problem in the transition segment by using DNN to generate a smooth output feature that outputs the entire wideband spectrum instead of just predicting the high-frequency spectrum [15]. When the data length is very long, the first data and the last data have little correlation. Because the output generated by DNN is too smooth, although the high-frequency information of narrowband speech is roughly recovered, the high-frequency spectrum is blurred and lacks a lot of detailed information and texture structure when observed from the speech spectrogram, which shows a weakness in the high-frequency spectrum recovery.

The neural network model proposed in this paper is mainly based on time-domain waveform modeling, so the model's ability to model time-series data directly affects the quality of reconstructed wideband speech, and how to introduce a time-series feature extraction factor into the model becomes a factor worth considering. The model learns the nonlinear mapping relationship between narrowband speech to wideband speech, and the model's ability to learn and characterize the nonlinear mapping relationship is also worth studying. In addition, the input dimension of the time-domain speech waveform as a model is too high, even if the input is a frame of speech waveform, the dimensionality will cause the model to be too complex, so how to reduce the model complexity of modeling the time-domain speech waveform will also be a research focus.

## 3. Depth Neural Network Algorithm Design

As the core technology of artificial intelligence, scientists have achieved many successes in the direction of machine learning, but at present, when applying machine learning algorithms in related fields, it often takes a long time to design input feature representations by hand, and whether these features can be selected well depends largely on experience and luck [16]. In addition, most traditional machine learning algorithms use relatively simple network structures, which can only uncover shallow information in the data, and they are very limited when faced with complex tasks and applications. Deep learning algorithms have emerged to solve these problems. The aim is to build neural networks that mimic the mechanisms of the human brain, enabling machines to interpret, analyze, and learn from various types of data just like humans do, and to access the underlying patterns and levels of expression contained in the samples. In the process of gradient backpropagation, RNN is prone to gradient disappearance and gradient explosion, which makes RNN unable to learn the relationship between long-distance sequences. The biggest difference between deep learning and shallow learning lies in the depth of the structure; the former usually has five, six, or even more than ten hidden layers, which can effectively make use of complex, combinatorial nonlinear functions to obtain the distributed features of the data at a deep level. Ultimately, deep learning is a complex machine learning algorithm that performs far better than previous related techniques in many artificial intelligence tasks.

**Figure 1:**
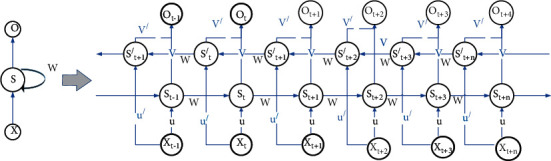
RNN time dimensional expansion diagram.

A recurrent neural network (RNN) is designed to specifically process temporal data, such as speech, video, and text. The basic recurrent neural network structure is shown in Figure 1, where the input *x* is passed through the hidden layer *S* to obtain the output *O*. The right side of Figure 1 shows the expansion of the recurrent neural network in the time dimension, showing the RNN states at three time steps. The output of the recurrent neural network not only depends on the input at the current moment but also considers the input at the previous time step. The formula is expressed as

(1) $$\begin{array}{c} O_{t}=VS_{t}+VS_{0}, \end{array}$$

(2) $$\begin{array}{c} S_{t}=Ux_{t-1}-WS_{t}, \end{array}$$

(3) $$\begin{array}{c} O_{t}=V\left(Ux_{t}\right)-W\left(Ux_{t-1}+Ux_{t-2}+\ldots \right). \end{array}$$

As we can see from equation (3), the output of the recurrent neural network *O*_t_ at the current moment is influenced by {*x*_*t*−1_, *x*_*t*−2_, *x*_*t*−3_…}. Since the RNN has only one state, it is more sensitive to the neighboring data. Although the whole temporal data is considered, when the data length is long, the first data and the last data are not much related to each other. Moreover, RNN only considers the temporal data before the current moment, but not the data after the temporal data, which is still lacking in the learning ability of contextual relationships. Moreover, the RNN is prone to gradient disappearance and gradient explosion in the process of gradient backpropagation, which leads to the inability of the RNN to learn the relationship between long-range sequences.

Before a deep learning model is put into real-life use, it needs to be trained first. Before that, it needs to be initialized; that is, given initial values of the model parameters, and in the training process, the training data are input to the model, and after the initialized model, the predicted values are output. The difference between the predicted value and the labeled value is called the error: the larger the error, the less the predicted value is from the labeled value, the smaller the error the more the predicted value and the labeled value is like; the purpose of training the model is to expect the predicted value and the labeled value to be the same, the ideal situation error value is 0. The labeled value is fixed, only by changing the predicted value to converge to the labeled value, thus reducing the error. The prediction value is generated by the model, and the parameters of the model determine the prediction value generated by the model. The final thing that needs to be changed is the model parameters. In general, using the distance formula to define the predicted value and the label MSE also known as *L*2 objective function, the mean of the square of the difference between the predicted value and the label value is calculated and the MSE is defined as follows: the error between the values, the gradient of the error is calculated, and the gradient is backpropagated to update the model parameters.

(4) $$\begin{array}{c} \text{MSE}=\sum_{i=1}^{N}{\left({\overset{\wedge }{x}}_{{}_{i}}-x_{i}\right)}^{2}. \end{array}$$

Since the length of the input sequence is different for each batch, we need to subject the input sequence to a sequence alignment operation, specifically by padding the shorter sequences with 0. But if the input sequence is too long, the left side is intercepted and the excess is discarded directly. This is done by adding a very large negative number (negative infinity) to the value of these positions, so that, after softmax, the probability of these positions will be close to 0.

In addition to the main encoder and decoder, there is the part of data preprocessing, which discards RNN, whose biggest advantage is the abstraction of data on time series, so it is necessary to transfer the position information to the encoded part by the position encoding method. In this thesis, the sine and cosine functions will be used to construct the value of each position, which is constructed as shown in the following equation

(5) $$\begin{array}{c} PE\left(\text{pos},2i\right)=\text{cos}\left(\frac{1000^{d_{\text{mod}el}/2i}}{\text{pos}}\right). \end{array}$$

Multidecimal quantized speech signals need to be transmitted, stored, and calculated on electronic devices and are first binary encoded using a bit depth that represents the number of bits of information in each sample point, typically 8, 16, 24, and 32 bits for microphones. The quantization bit depth of 8 bits is usually used, so the quantization bit depth of the microphone used to capture speech during a cell phone call is also usually 8 bits.

**Figure 2:**
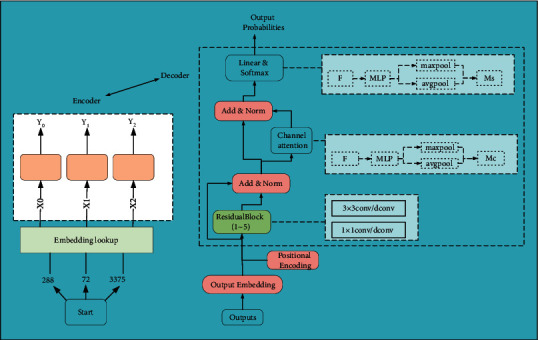
Schematic diagram of the model structure.

Given that STFT is also a linear operation, in some deep learning-based speech tasks, modeling the time domain waveform directly yields more optimistic results than the frequency domain. Therefore, STFT processing of speech in the preprocessing stage of speech band extension is no longer a necessary operation, and the model is directly allowed to learn the nonlinear mapping relationship between narrowband and wideband speech, and the model itself is allowed to extract useful features to recover high-frequency information [17]. Because the Fourier transform is very difficult to learn, the speech time-domain waveform has higher dimensionality compared with the frequency domain features, which requires more learning ability of the model itself, and the model needs to increase the number of parameters and complexity to learn the nonlinear mapping relationship between narrowband and wideband speech waveforms, as shown in Figure 2.

The lexical recognition score refers to the mean reaction time, standard deviation, and accuracy of the word when acting as a stimulus word in the lexical judgment and naming tasks. The reaction time in the lexical judgment task refers to the time it takes for the subject to determine whether the target word is a true or false word in English; the reaction time in the lexical naming task refers to the time it takes for the subject to pronounce the target word. The response times and accuracy rates of native English speakers on 40,481 words in the lexical judgment and lexical naming tasks were recorded in the ELP database, and TABLES 2.0 used them as the basis for calculating eight indicators for the lexical judgment and naming tasks. As the number of training steps increases, uniparameter migration, parameter migration, and parameter migration + BPE all show an increasing trend. When the number of training steps reaches 80,000, the target BLEU value is reached.

**Figure 3:**
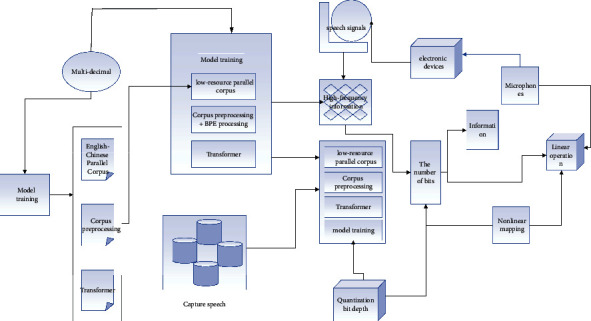
System model migration training diagram.

ANOVA requires two tests; the first test lets us know if there is a difference between the means of three or more groups, and the second test lets us determine exactly between which paired groups the difference occurs. The first test is called the omnibus test or integrated *F*-test and is shown in Figure 3 if a difference is found. The variance test does not tell us exactly where the differences occur; is there a difference between group 1 and group 2 or group 3? This requires a second test to determine exactly where the difference lies. The second test is multiple comparisons, and there are two types of multiple comparisons: one is a priori or planned, or ex-ante test, which is mostly used in validation studies because the researcher has anticipated or assumed which group differs from which group before collecting the data. The researcher has his or her research hypothesis based on the theory in question, and the use of prior testing is only to test his or her hypothesis.

To make a fair comparison in all experiments, for each translation task, the neural machine translation method based on transfer learning uses a neural machine translation model trained in a large English-Chinese corpus environment for transfer learning and training. The case-sensitive BLEU value is used as the evaluation index of the translation effect in all experiments. Specifically, the BLEU value is calculated using the multi-BLEU. Perl script and a larger BLEU value mean a better translation effect. In the testing phase, for the Transformer model, as in previous related studies, the experiments are set beach-size to 4. To verify the effectiveness of the Transformer framework for neural machine translation based on migration learning, in this section, several control models are compared for the experiments. The experimental results show that the model migration learning proposed in this chapter can effectively alleviate the problems existing in low-resource neural machine translation. In other words, when the noun phrase contains more meaning components, the corresponding text complexity also increases. The higher the score of a text on this principal component, the more complex the expansion of noun phrases in the text, the greater the amount of information embedded in the noun phrases, the higher the sentence complexity, and the more complex and difficult the text is to understand.

## 4. Experimental Design for Analysis of Factors Associated with the Improvement of English Translation Ability

For most of the text detection models, they are usually only applicable to images where the text lines are close to the horizontal direction. However, in real life, the angle of the pictures taken by people through cell phones and other tools is more diverse, and the orientation of the text cannot be guaranteed, so the positioning results obtained by inputting them directly into the text detection model may not be accurate.

In the menu images, most of the text that appears is the names of various dishes and the corresponding prices. Considering that most of the prices are Arabic numbers, the accuracy of recognition is high, and no subsequent error correction is needed, so only the information of dish names needs to be collected. Through web search and website crawler technology, this project collected about 1900 Chinese dish name data and about 1400 English dish name data.

(6) $$\begin{array}{c} \text{Precision}=\frac{\text{FP}+\text{TP}}{\text{TP}}\text{,}\\ \text{Recall}=\frac{\text{TP}-\text{FN}}{\text{TP}}\text{,}\\ F_{1}=2\left(\text{Pecision}-\text{Recall}\right)-\text{Pecision}\times \text{Recall}. \end{array}$$

In addition, 3 features of noun superordination, 2 specificities, and 2 imagery of text real words and all words were negatively loaded into this principal component. The higher the mean value of the superordination of all nouns in the text, the lower the text score on this principal component. The higher the superordination of nouns, the fewer nouns with general concepts and the more nouns with concrete meanings in the text, and the more specific the nouns are. Concreteness refers to the degree of nonabstraction of the vocabulary; the more specific the vocabulary in the text, the more easily the text vocabulary is understood. Similarly, the degree of imagery refers to the ease of constructing the imagery of a word, and the easier it is to imagine a word, the higher the degree of imagery. If the text contains more words with higher concreteness and imagery, the lower the score of this principal component of the text. Language barriers are increasingly severe and obvious, and the need for seamless communication and understanding becomes critical.

**Figure 4:**
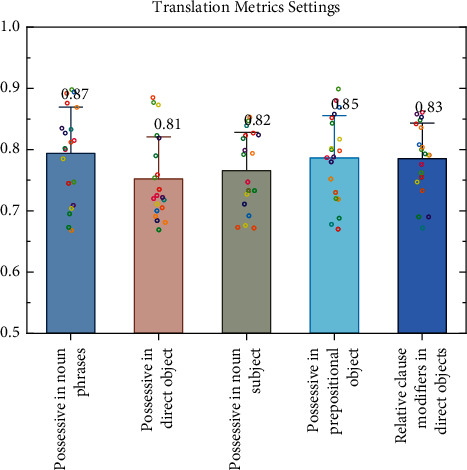
Translation index settings.

As shown in Figure 4, the last component describes the verb specificity, which mainly describes the superordination value of verbs in WordNet-based target texts under different calculation methods [18]. The lower the level of a verb in the WordNet hierarchy, the higher the superordination value of the verb and the more specific the meaning of the verb; and the higher the level of a verb in the hierarchy, the more specific and meaningful the verb is. If a text has a higher score for this principal component, it means that the text contains fewer general meaning verbs and more specific meaning verbs. It has been shown that as language proficiency increases, learners acquire more specific meanings of words, and these words have more superlatives; thus, the value of superlatives is proportional to *L*2 writing level.

The frequencies of the active word lexical items in the VAC contained in the text in all COCA written language bases and the frequency of class only characters, the log-transformed values of the frequencies of the VAC in the text in all COCA written language bases, and the standard deviation of the frequencies were negatively transferred to this principal component. With the improvement of language level, the meaning of vocabulary mastered by learners is more specific, and these words have more synonyms. Therefore, the value of synonymy of vocabulary is proportional to the level of *L*2 writing. The log-transformed form of frequencies is a log-transformation of the raw mean values, thus better matching the characteristics of the Ziff distribution of the data. The class character only indicator is a count of only the number of different main word lexical items. A text that scores higher in this component indicates that the more frequently the main verb lexical items, main verbs, and VAC combinations in the text are used in all COCA written corpora. The ratio of VACs in a text that appears in the COCA written corpus to all VACs was also positively loaded into this principal component; that is, the more VACs in a text that contain occurrences in the COCA corpus, the higher the score of this principal component.

This component describes the overall number of clauses containing subordinate components, the use of relational clauses as modifiers in noun phrases and prepositional objects, and the number of prepositions and conjunctions contained in the clauses; TAASSC treats both qualifying and nonqualifying clauses as clauses in the index calculation [19]. Because the output generated by DNN is too smooth, although the high-frequency information of the narrow-band speech is roughly restored, the high-frequency spectrum is blurred from the spectrogram, and it lacks a lot of detailed information and texture structure. The more subordinate clauses the text contains, and the more relational clauses act as modifiers in noun phrases and prepositional objects, the higher the text score for this main component. In addition, this component also includes the variation in the number of subordinate components in clauses, which is mainly reflected in the standard deviation indicator; that is, the more the number of subordinate components in small clauses and the greater the variation in the number of subordinate components in different clauses, the higher the test scores on this main component.

**Figure 5:**
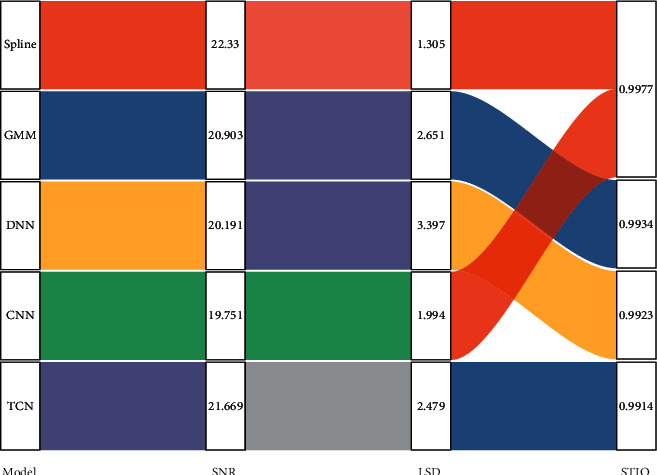
Objective evaluation results on the dataset.

For a fair comparison, the basic Transformer model is used in all experiments, the word embedding dimension is set to 512, the denoising self-encoder uses a Transformer structure based entirely on the attention mechanism, the number of encoder layers is 6, the number of decoder layers is 6, the number of hidden units in the fully connected layer is 512, and the encoder state is used to initialize. The decoder is initialized with the encoder state. The model is based on the PyTorch open-source neural machine translation system. The English-Chinese translation model and the low-resource translation model are both Pytorch/nmt, and the encoder and decoder of the model both contain residual linking and regularization. To solve the problem of off-table words, a byte-pair encoding method is used to divide words into subword units using 3200 BPE operations, as shown in Figure 5.

This paper proposes a new type of encoder and decoder network, which retains the advantages of the original encoder and decoder network, and adds aurous convolution to the encoder and decoder, thereby increasing the receptive field and enhancing the model's contextual learning ability for time series data. Thanks to the continuous development of the Internet and artificial intelligence, combining text recognition technology and machine translation technology will be able to solve this real problem for us very well. Although there are many open platforms for text recognition or text translation launched by large companies in the market, they are all oriented to general-purpose fields, and the effect of using them in complex scenarios plummets and lacks applicability. Therefore, this topic is dedicated to building a text recognition and translation system with good performance in vertical fields to facilitate our daily life.

## 5. Analysis of the Performance Results of the Deep Neural Network Algorithm

To ensure that the indicators included in each principal component are correlated, the absolute value of the small coefficient of the cancellation eigenvalue is set to 35. If an indicator is included in more than one principal component, we only take the component in which its highest loadings are located to have eigenloadings of 82 and 43 in component 1 and component 2, respectively, both of which are higher than 35, and we only keep it in component 1 because its eigenloadings in that component are much higher than the loadings in component 2.

The students with a weaker speaking foundation have more prominent problems in vocabulary pronunciation. Therefore, in this round of practice, the author and the teacher paid more attention to the students' vocabulary pronunciation problems, picked out the problems of word pronunciation in the students' dubbing works, and let the students correct them during the repeated dubbing to help them improve their speaking ability [20]. While students are dubbing, they are implicitly enriching their vocabulary and their translation skills can be improved to a certain extent. Because the subtitle displays in the dubbing resources are in both English and Chinese, students' repetitive imitation of the original voice in the dubbing is conducive to effective memorization and understanding of English and Chinese sentences and English words. Memory and imitation are very important in language learning and can effectively turn short-term memory into long-term memory and store it. Blind voice band expansion, that is, at the receiving end of the communication device, the narrowband voice is processed and restored through the voice band expansion algorithm. Blind voice band expansion does not require changing the existing communication network channels and voice acquisition methods and will not cause additional economic burden.

**Figure 6:**
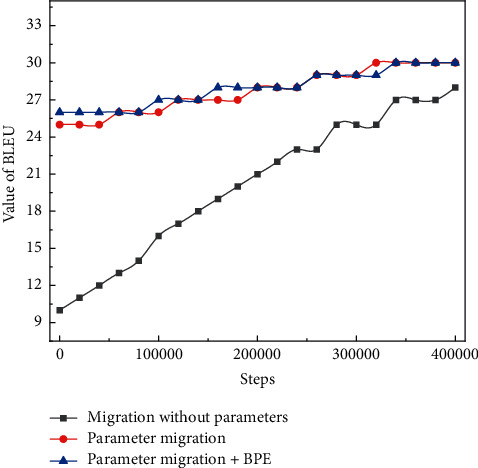
Relationship between low-resource Chinese-English BLEU values and training steps.

By comparing the experiments in Figure 6, it can be concluded that the results (BLEU values) of English neural machine translation using model migration are better than the results of traditional machine translation without using migration learning strategy. Trained based on large-scale Chinese-English model training migration, the BLEU values improve faster in the early stage than the translation models that rely entirely on the English corpus for neural network initialization. With a target point of BLEU value of 25 for the Chinese-English low-resource parallel corpus, the translation model with model migration can be trained to 20,000 steps, but the traditional translation model without model migration needs to be trained to 80,000 steps. The low-resource parallel corpus is trained to a BLEU value of 40, and the translation model with model migration can be trained to 50,000 steps, but the traditional translation model without model migration needs to be trained to 80,000 steps.

Because of parameter initialization before the training of the machine translation model, the parameters of the large-scale Chinese-English translation model belonging to the same translation task are introduced into the initialization of the low-resource Chinese-English and Tibetan-Chinese translation models so that the model already has a certain parameter base before training, so its learning rate will be improved when retraining. The attention mechanism gives the network the ability to reconsider all input words and use this information when generating new words. The previous architecture was redesigned with a convolutional neural network that processes all input words together, thus making the training and inference process faster.

**Figure 7:**
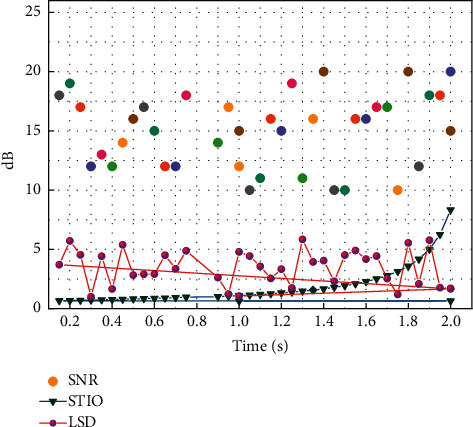
Objective evaluation results on the TIMIT dataset.

Figure 7 shows the change of BLEU values with the change of migration times using data migration in this section. Through the comparison experiment in Figure 7, we can analyze that the results (BLEU values) of Chinese-English and Tibetan-Chinese neural machine translation using data migration are better than the results of traditional machine translation without using model migration learning strategy. Meanwhile, we can find that in the case of data migration, the BLEU values of 33.64 and 51.42 are the best test results for Chinese and English low resources, in which the BLEU values of the translation models both take the BT4 translation model as the turning point, and their model translation results reach the best when we go through 4 times of data migration.

And the perceptual domain loss function in the time-frequency perceptual loss function also only relies on the log-Meier spectrum for the initial model to learn the perceptual domain features and cannot further enhance the model to distinguish between reconstructed wideband speech and wideband speech. Machine translation technology has become a research hotspot in the field of information processing, and its purpose is to reduce the language barriers between people in different countries and different nations through machine translation. Therefore, a new codec network is proposed in this chapter, which adds a cavity convolution to the codec while retaining the advantages of the original codec network, thus increasing the perceptual field and enhancing the model's ability to learn the context of the temporal data. In addition, on the temporal-frequency perceptual loss function, this chapter uses the perceptual metric based on the deep learning model as the perceptual domain loss function, which has good auditory perceptual characteristics and has a strong ability to discriminate differences. The experiments show that the method proposed in this chapter achieves better results on both subjective and objective evaluation experiments.

## 6. Analysis of Experimental Results

**Figure 8:**
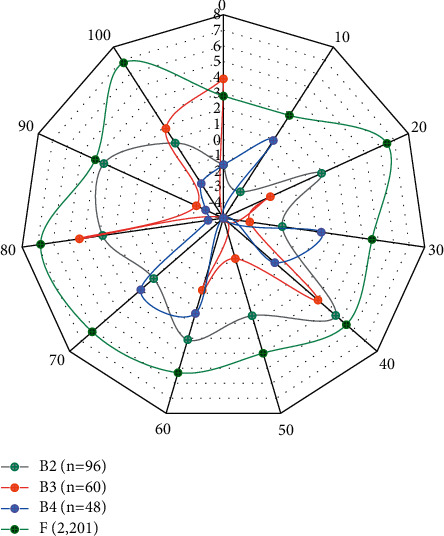
Complexity metric book-level differences.

The average number of prepositions in the sentence is 1/4 = 0.25, the average number of adjective modifiers in the noun phrase is 2/4 = 0.5; the average number of qualifiers in the noun phrase is 4/4 = 1; the average number of subordinate components in the noun phrase is 7/4 = 1.75; the average number of subordinate components in the noun subject is 1/1 = 1; the average number of subordinate components in the prepositional object is 2/1 = 2; and the number of adjectival modifiers indirect objects was 0/1 = 0. As learners develop their language proficiency, they tend to use more complex noun phrases more often, and noun phrases contain more subordinate components. In other words, when noun phrases contain more meaningful components, the corresponding text complexity increases. The higher a test scores on this main component, the more complex the noun phrase expansion in the text is, the more information embedded in the noun phrase, the higher the sentence complexity, and the text is thus more complex and less understandable, as shown in Figure 8.

**Figure 9:**
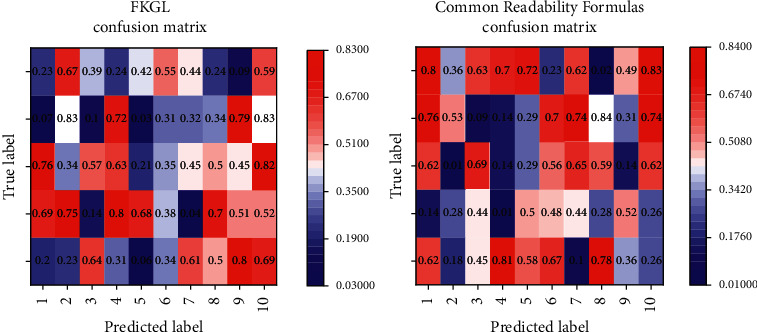
Predicted label confusion matrix for model classification results.

The goal of translation ability research is to break through this predicament, clear the fog for translators, clarify the components of translation ability, better guide translation practice, and promote the improvement of translation quality. In this study, the single-layer feedforward backpropagation neural network in the net package is used to construct the model, whose basic parameters include the number of neurons in the hidden layer (size), the initial random weights (rang), the decay parameter of neuron input weights (decay), and the maximum number of iterations (max). The parameters are configured as follows: firstly, the initial random weights and the maximum number of iterations are determined based on experience and the development manual of the nnet package, which are 0.1 and 1100, respectively; secondly, the number of neurons and the decay parameter of the weights are automatically tuned using the tuning grid. After automatic model tuning and comparison, the final decay value was determined to be 0.1 and the number of neurons was 27, as shown in Figure 9.

FKGL, which has the strongest predictive power in the readability formula, has prediction accuracies of 84%, 52%, and 81% on the three levels, respectively. This is because the average sentence length (number of words) and word length (number of syllables) are the basic parameters of the FKGL formula, and these superficial features can distinguish well between texts of low or high complexity, while texts of medium complexity cannot simply rely on superficial features such as sentence length and word length. Moreover, even if we include all the above seven readability indicators into the modeling features, the prediction accuracy of each level does not improve and even decreases in the low and medium complexity levels.

The focused text features are also at the lexical and syntactic levels, based mainly on the analysis of indicators such as word frequency and average sentence length in the target text, which are applied to a linear readability formula and subjected to logarithmic processing and standard deviation analysis to obtain the difficulty value of the target text. This study partially corroborates the previous view that vocabulary and syntax do have an impact on text complexity, but both lexical complexity and syntactic complexity have low accuracy in predicting intermediate complexity levels. In addition, we found that the syntactic complexity model has the lowest recall (*r* = 56.69%) and high standard deviation (SD = 9.19) among the various types of features, indicating that the model is based on syntactic complexity features is highly volatile and less stable. 

## 7. Conclusion

This study aims to construct a text complexity hierarchical model with the help of a deep neural network approach. Through the study of various neural machine translation methods, we found that obtaining a high-quality pretrained model largely influences the translation effectiveness of a neural machine translation model when initializing the model using pretraining deep learning techniques, because pretraining is a network that has been trained and saved, which has been previously trained on a large dataset, we can use the pretrained model as a feature extraction device for migration learning. after the dimensionality reduction of the 3-dimensional indicators, a total of 5 principal components of lexical complexity are extracted, which cumulatively explained 85.7% of the variance of the data. These 5 lexical dimensional principal component features are, in order, lexical frequency and distribution, academic written language features, ternary word mutual information, binary word frequency and distribution, and verb specificity; 8 principal components were extracted for syntactic complexity, which cumulatively explained 67.6% of the total data variance. Migration learning can be used better when the features learned by the pretrained model are easy to generalize. Then, the data migration idea is used to expand the data for the low-resource parallel corpus separately. Finally, the effectiveness of using the data migration algorithm in this paper is verified using experiments, and the model tested with data migration obtains higher accuracy compared with other translation models without introducing data migration. The data expansion process is repeated until its translation performance no longer improves, and the experimental results show that the model translates best after four data migration experiments.

## Data Availability

The data used to support the findings of this study are available from the corresponding author upon request.
